# Civilization in Its Relation to the Increasing Degeneracy of Human Teeth

**Published:** 1882-01

**Authors:** 


### CIVILIZATION IN ITS RELATION TO THE INCREASING DEGENERACY OF HUMAN TEETH.

Dr. Norman Kingsley, of New York, read a paper at the International Congress on this subject. He said:

The most important undetermined problem now confronting the Dental profession was embodied in the inquiry made daily by anxious parents in substantially the following form:—'Why do my teeth decay more rapidly than my father's or mother's did, and why are my children's teeth decaying at an early age than mine?' The inquiry did not come from those who neglected their teeth, or from the lower classes of society, the ignorant or the depraved. It was confined to no particular race or nationality, but came from that class which was the most intelligent, the most highly cultured, and the most finely organized in any community, irrespective of race, locality, or climate. It was useless to treat the inquiry lightly, or to attempt a denial of the premises. The cases were exceedingly rare, if they existed at all, where the teeth of the children were sounder than those of the parents, and we must admit the conclusion that with each succeeding generation the dental organs were becoming more and more degenerate. What response had the practitioner to this inquiry? It was not difficult to formulate an answer which would satisfy many. Some even who were well versed in other branches of science would often be satisfied with an answer that would not bear investigation, and might even be absurd. In this way was heard daily a repetition of theories and speculations which had just enough foundation of truth, and just enough plausibility, to escape being challenged. Probably the most generally received idea amongst cultivated unprofessional people was that the eating of candy and other sweetmeats was the cause of dental caries. There was probably no more fallacious idea so generally believed; pure candy in moderate quantity never harmed any healthy child or adult. Another speculation which had gained some credence was contained in the discovery that iced water, hot drinks, or hot cakes were the cause of all the mischief; but he believed it to be very doubtful whether a cavity of decay ever originated from sudden changes of temperature. That decay was caused by living upon soft food instead of upon food that required mastication, was another favorite theory. The nature of the food had unquestionably a most important influence upon the dental organs, and might indirectly cause decay, but the use of soft food was not the primary cause of the present increasing degeneration of the tooth structure, as would be evident from the fact that the Esquimaux, living on whale blubber, the Chinaman, living on boiled rice, and the savage, who lives on nuts and fruits, have all equally good teeth, though those of the latter are worn down more quickly.

Still another theory was embodied in the statement that contact always produced decay. That contact was anything but an incident to the real cause could not be shown. An examination of the most solid dental structures ever presented for professional inspection showed all the teeth in absolute contact, with no traces of decay. The contact theory must be relegated to its proper place among secondary causes, simply as a coincident factor in the great problem. Another theory assigned the want of cleanliness as the cause. This also was only a factor which must be associated with many others to give it any potency. We frequently saw perfect dental structures which had never known cleanliness as at present understood. Cleanliness was more important in its remedial character than as an explanation of the primary cause. 'Climatic influences are the cause,' said another, and still another maintained that the cause was intermarriage, or the mixing of types. Certain districts might be malarious to the unacclimatized, and the whole system might be poisoned, but tooth structure suffered, if at all, only as a secondary result. Interbreeding and crossing tyes of the human race ought not to result in defective tooth structure. Reasoning from analogy with regard to what took place in the case of animals, improvement should be looked for, but interbreeding in its highest application involved that only sound and healthy subjects should be concerned. The mixing of inharmonious types was more likely to result in deformity than in deterioration of structure. Other vague theories, used often without much understanding, were formulated, but the most comprehensive, and the most tangible withal, which had been made sponsor for this growing curse of the human race was *civilization.* What was civilization, and what had civilization to do with decaying teeth? It meant, an emerging out of barbarism into refinement, out of ignorance into knowledge, out of bondage into liberty, out of privation into comfort. Civilization expands the intellect, represses vice and savage instincts, cultivates virtue and noble aspirations, encourages the growth of the emotional nature, and enlarges the domain of human sympathy. Civilization defies and controls the elements, organizes commerce, builds cities, railways, telegraphs and factories. Civilization is the divinely appointed method through which mankind derive their greatest blessings, and by which they reach their highest possible state of intellectual, moral, and social development. Only through civilization would the millennial or ideal existence of the human race ever be attained; civilization, therefore, was a normal condition of mankind. In the more refined and luxurious conditions of life we found physical labor exchanged for mental, and strain of mind took the place of strain of body. Muscular tension ceases, and nervous tension takes its place. The mind is constantly on the alert, and the brain has no rest. The nutrition of muscles and bones is diverted to repair the undue waste of nervous tissue, and, sooner or later, come, inevitably, the long list of nervous diseases, which now so threateningly confront us. The causes which tend to produce such results were more active and more potent in the Northern United States than in any locality in the globe. The reasons for this had their foundation primarily in the institutions of the country, and the stimulus which the country afforded for the intensest mental activity. But testimony was not wanting that the same thing in kind was now going on in Great Britain, and even heretofore stolid Germany was undergoing a like transition. Nervous diseases and decay of teeth were correlated, both being symptoms of a common cause. The teeth required constant nutrition, as did the muscles, the bones, or any other organs or tissues of the system. Teeth decay primarily because the nutrition of their organic structures being withdrawn, retrogade metamorphosis ensues. Caries is simply solution or disorganization of both constituents by agents which are always external, but which would be quite inert under other constitutional conditions. When nutrition is insuffïcent or diverted, the resisting power of the vitality inadequate, and destructive agents present, the teeth will yield at their weakest point, and caries is the result. The ordinary remedy for these evils, and the salvation of the race from degeneracy and destruction would seem to involve a return to a condition of life more consistent with hygienic laws. The pessimistic view of the future was without reason, for while degeneracy of the teeth was certainly on the increase in certain families and classes, there were equally certain signs of its abatement. With increasing wealth, families had less cares, less anxiety, less nervous strain, more ease, more attention to hygiene, and better habits of life. The intellectual activity of to-day, and the energy and intensity of modern thought were not inconsistent with a sound constitution, with perfect health in all the tissues, and with long life. It was the worry which wears out the nervous system, and not the work. Civilization, the glory of mankind in their maturity, is, nevertheless, in no wise responsible for the accidental effects which have resulted from a violation of her true principles, for out of civilization ought to, and must come, the grandest examples of humanity the world has yet seen, without spot, or blemish, or taint of disease.—*Jour. Brit. Dental Association.*

